# Detection of *Borrelia burgdorferi sensu lato* and spotted fever group rickettsiae in hard ticks (Acari, Ixodidae) parasitizing bats in Poland

**DOI:** 10.1007/s00436-016-4936-2

**Published:** 2016-02-02

**Authors:** Krzysztof Piksa, Joanna Stańczak, Beata Biernat, Andrzej Górz, Magdalena Nowak-Chmura, Krzysztof Siuda

**Affiliations:** Department of Vertebrate Zoology and Human Biology, Institute of Biology, Cracow Pedagogical University, Podbrzezie 3, 31-054 Kraków, Poland; Department of Tropical Parasitology, Medical University of Gdańsk, 9B Powstania Styczniowego str., 81-519 Gdynia, Poland; Department of Invertebrate Zoology and Parasitology, Institute of Biology, Cracow Pedagogical University, Podbrzezie 3, 31-054 Kraków, Poland

**Keywords:** Bats, ticks, Ectoparasites, *Borrelia burgdorferi* s.l., SFG *Rickettsia*, Poland

## Abstract

A total of 491 *Ixodes vespertilionis* and 8 *Ixodes ricinus* collected from bats and cave walls in southern Poland between 2010 and 2012 were examined by the polymerase chain reaction for tick-transmitted pathogens. PCR analysis for *Borrelia burgdorferi* s.l., *Rickettsia* spp., and *Anaplasma phagocytophilum* yielded negative results for all *I. vespertilionis*. DNA of *Rickettsia helvetica* was detected in three specimens of *I. ricinus* attached to *Rhinolophus hipposideros* or *Myotis myotis*, while *Borrelia garinii* was found in one tick parasitizing *Myotis daubentonii*. These pathogens were recorded for the first time in hard ticks that parasitized bats.

## Introduction

Multiple sources report that ticks are reservoirs and vectors of a variety of pathogens (Jongejan and Uilenberg 2004). Of 78 tick species recorded in Europe (Nowak-Chmura and Siuda 2012; Hornok et al. 2014) five hard ticks (*Ixodes vespertilionis*, *I. simplex*, *I. ariadnae*, *I. ricinus* and *I. trianguliceps*) and one soft tick (*Carios vespertilionis*) were recorded on bats (Haitlinger 1978; Hornok et al. 2014; Siuda et al. 2009; Ševčík et al. 2010). Of these, only *I. vespertilionis*, *I. simplex*, and *I. ariadnae* are specific parasites of bats. They mainly inhabit caves and cave-like shelters (Filippova 1977; Hornok et al. 2014; Siuda et al. 2009; Ševčík et al. 2010). In contrast, *Ixodes ricinus* and *I. trianguliceps* are exophilic species that parasitize a wide range of vertebrate hosts (Eisen and Lane 2002; Hillyard 1996) which occasionally includes bats (Piksa et al. 2014; Siuda et al. 2009; Ševčík et al. 2010). Both species are the important vectors of pathogenic agents in Europe (Bown et al. 2008; Gray 1998; Süss et al. 2008).

Chiroptera are the second most specious mammalian order next to rodents; currently, over 1300 species are known (Fenton and Simmons 2015). However, despite the high species richness of bats, there are almost no reports on vector-borne agents occurring in ticks parasitizing these mammals. So far, *Borrelia* spp., including *Borrelia burgdorferi sensu lato*, *Rickettsia* spp., and *Ehrlichia* spp. have been found in the soft tick *C. vespertilionis* (also known as *Argas vespertilionis*) (Hubbard et al. 1998; Socolovschi et al. 2012), while *Rickettsia* spp., *Borrelia* spp., and *Bartonella* spp. were detected in *Carios kelleyi* (Loftis et al. 2005). On the other hand, of hard tick species associated with bats, only in *I. vespertilionis* such pathogenic agents as *Bartonella* spp. were found (Hornok et al. 2012).

Results of recent studies indicate that immature and adult stages of ticks are being frequently found on bats in Poland (Piksa et al. 2013; Siuda et al. 2009). These ticks potentially may serve as vectors of different pathogenic agents. Thus, the purpose of our preliminary study was to analyze ixodid ticks collected from different bat hosts for the presence of pathogenic *Borrelia* spp., *Rickettsia* spp., and *Anaplasma phagocytophilum*.

## Materials and methods

### Tick collection

Ticks were collected from (1) bats captured outside the caves into mist-nets and harp-traps during post-hibernal, summer, and swarming activity; (2) during hibernation period through direct collection from bats and from bats captured before the cave entrance; (3) handled from cave walls; and (4) during survey of bats in shelters of nursery colonies. Samples were collected between 2010 and 2012 in a dozen localities in the Polish Carpathians and Krakowska-Wieluńska Upland (Table 1). The fieldwork was carried out under permits issued by the Polish Ministry of Environment and the General Directorate for Environmental Protection (DLOPpn-4102-517/24815/10/RS, DOPpn-4102-705/35666/11/RS, DLOPpozgis-4200/VI.D-3/1263/10/km, DOP-OZGIZ.6401. 09.18.2011.km.2).

**Table 1:** The number of ticks examined for the presence of tick-borne pathogens

Tick species	Host species	Number of collected specimens:				
		Larvae	Nymphs	Females	Males	Total
*Ixodes vespertilionis*	Cave wall		42	75	46	163
	*Rhinolophus hipposideros*	126	137	46		309
	*Myotis myotis*	1	1	1		3
	*Myotis nattereri*	6				6
	*Myotis emarginatus*	6	2			8
	*Myotis brandtii*			1		1
	*Myotis mystacinus*		1			1
Total		139	183	123	46	491
*Ixodes ricinus*	*Rhinolophus hipposideros*			4		4
	*Myotis myotis*			1		1
	*Myotis bechsteinii*	1				1
	*Myotis daubentonii*			2		2
Total		1	0	7	0	8

All ticks were stored in 70 % ethanol for morphological examination and subsequent molecular studies. Ticks were identified to species and developmental stage (larvae, nymphs, males, and females). The taxonomic identification of ticks was done using a light microscope by comparison with characteristics presented in taxonomic keys and with description of species found in papers by Siuda (1993), Arthur (1956) and Manilla (1998).

### DNA extraction and PCR analysis

The total DNA was extracted from individual unfed ticks by lysis in ammonium hydroxide (NH_4_OH) (Rijpkema et al. 1996) and from ticks of different stages of engorgement by using Sherlock AX commercial kit (A&A Biotechnology, Gdynia, Poland). The obtained lysates were kept at −20 °C. Regular, semi-nested and nested PCRs were performed in order to detect spirochetes of *Borrelia* spp., rickettsiae *A. phagocytophilum*, and *Rickettsia* spp.

A nested PCR was conducted to detect for the presence of *Borrelia* spp. within individual ticks. The protocol of Wodecka et al. (2009) was used to amplify 774- and 605-bp fragments of the *fla* gene, using primer pairs 132f and 905r, and 220f and 824r, respectively.

Another nested PCR assay was performed for the detection of *A. phagocytophilum* DNA. Primer pairs ge3a and ge10r, and ge9f and ge2, and the protocol of Massung et al. (2002), were used to amplify a 546-bp fragment of the bacterial 16S rRNA gene.

Rickettsial DNA was detected by a regular PCR using primers RpCs.877p and RpCs.1258n, which amplify a 381-bp fragment of the citrate synthase gene (*gltA*) of *Rickettsia* spp. (Regnery et al. 1991). Then, positive samples were subjected to nested and semi-nested PCRs, designed to amplify a 355-bp region of the *OmpA* gene and 757-bp region of the 16S rRNA gene, respectively. Nested PCR primes SLO1F/SLO1R (outer) and SLO2F/SLO2R (inner) as well as semi-nested primers Ric, Ric U8, and Ric Rt were used as previously described by Raoult et al. (2002) and Nilsson et al. (1997), respectively.

Each PCR reaction was performed in a reaction volume of 20 μL containing 0.5 μL RUN Taq polymerase (1U/1 μL) (A&A Biotechnology, Gdynia, Poland), 2 μL 10× PCR Buffer (A&A Biotechnology, Gdynia, Poland), 2 μL dNTPs mixture (10 mM) (Fermentas, Lithuania), 0.4 μL of appropriate primers, 12.7 μL double distilled water (13.7 μL for semi-nested and nested PCR), and 2 μL of the processed tick sample or 1 μL of the obtained PCR product for the semi-nested and/or nested PCR. As positive controls served *B. afzelii-*, *A. phagocytophilum-*, and *R. raoultii-*positive tick samples from our previous investigations served as positive controls (Stańczak et al. 2004; Stańczak 2006). The negative control was sterile water (*aqua pro injectione*).

All reactions were carried out in the GeneAmp® thermocycler PCR System 9700 (Applied Biosystem 850, CA, USA). Amplification products were analyzed after electrophoresis in a 2 % agarose gel stained with Midori Green DNA Stain (Nippon Genetics Europe GmbH).

PCR-positive products were purified with the clean-up purification kit (A&A Biotechnology, Poland) and sequenced in both directions with an ABI 310 Genetic Analyzer (Applied Biosystems, Foster City, CA, USA) by using the BigDye Terminator Cycle Sequencing kit version 3.1. (Applied Biosystems, Carlsbad, CA, USA) and the same primer pairs as for regular [RpCS.877p and RpCs.1258n], semi-nested [Ric and Ric Rt], and nested [220f and 824r] amplification. All sequences were compared with the corresponding sequences deposited in GenBank using BLAST (www.ncbi.nlm.nih.gov.blast)

## Results

A total of 499 ticks, including 491 *I. vespertilionis* (long-legged bat ticks) and 8 *I. ricinus* (sheep ticks) were selected from a few thousand ixodid ticks collected from bats in Poland in 2010–2012 (Table 1). All ticks were examined individually by PCR for the presence of *Borrelia* spp., *A. phagocytophilum*, and *Rickettsia* spp.

None of the examined long-legged bat ticks was found to be infected with any of the searched pathogens. None of the *I. ricinus* specimens was found to harbor *A. phagocytophilum*.

In contrast, *Borrelia* DNA was detected in one *I. ricinus* female (Table 2) parasitizing *Myotis daubentonii*. Subsequent sequencing of the 562-bp amplicon of the *fla* gene (GenBank acc. no. KJ577820) revealed that it was most similar (99.8 %) to *fla* gene sequences of *Borrelia garinii* (GenBank acc.nos. KF836512, KF918608, JF828688).

**Table 2:** Tick-borne bacteria detected in *Ixodes ricnus* collected from bats

Tick species and stage	No. ticks tested	No./% of infected ticks		
		*Borrelia burgdorferi* s.l.	*Anaplasma phagocytophilum*	SFG *Rickettsia* spp.
Larvae	1	0/0	0/0	0/0
Females	7	1/14.3	0/0	3/42.9
Total	8	1/12.5	0/0	3/37.5

Two *I. ricinus* females (Table 2) from *Rhinolophus hipposideros* and one *I. ricinus* female from *M. myotis* were PCR-positive for the rickettsial *gltA* gene. The sequences were 100 % homologous to each other and to the *gltA* sequences of *Rickettsia helvetica* (GeneBank acc nos. JX627379, KF447530, KC007126, JX040636, and AM418450). The consensus sequence (370 bp) was deposited in GenBank under acc. no. KJ577821. Moreover, the three positive samples were re-run and specific fragments of 16S rRNA gene of *Rickettsia* spp. were successfully amplified and sequenced. Also these sequences were identical and they shared 100 % similarity to four of *R. helvetica* sequences available in GenBank: GQ413963; AF394904, AF394905; L36212. The consensus sequence (719 bp) was deposited in GenBank under acc. no. KJ577822. However, all three samples that were PCR-positive for the rickettsial *gltA* gene were negative for the *ompA* gene.

## Discussion

The results of this preliminary study showed that some ixodid ticks collected from bats in southern Poland were infected with two species of pathogenic bacteria: *B. garinii* of *B. burgdorferi sensu lato* complex and *R. helvetica* of the spotted fever group rickettsiae (SFG). Both agents were detected in *I. ricinus* which is an accidental ectoparasite of bats in contrast to *I. vespertilionis* being a specific parasite of these hosts.

The role of a long-legged bat tick *I. vespertilionis* as a vector and reservoir of bacterial, viral, or protozoan pathogens is almost unknown. Until now, from bacterial agents, only *Bartonella* spp. has been detected in free-living individuals collected from cave walls (Hornok et al. 2012). The absence of *Borrelia* spp., *Rickettsia* spp., and *A. phagocytophilum* in *I. vespertilionis* tested in the present study indicate that this tick species is of little epidemiological significance.

In contrast, *R. helvetica* of SFG and *B. garinii* spirochetes were detected in three and one, respectively, different females of *I. ricinus* detached from bats. The presence of these bacteria in sheep ticks is not surprising. Infection rate of questing *I. ricinus* with borreliae in different collection sites in Poland may even reach up to 58.3 % (Stańczak and Kubica–Biernat 1999). In Europe, the metaanalysis of the mean prevalence of *Borrelia* spp. infections in ticks indicates that the overall mean was 18.6 % in adults and 10.1 % in nymphs (Rauter and Hartung 2005). The obtained *fla* gen sequence differed by one nucleotide with, among the other, sequences of *B. garinii* isolate O2-27 from *I. ricinus*, Lower Silesia, Poland (KF836512), *B. garinii* strain J3-1F-IR from *I. canisuga* feeding on a red fox *Vulpes vulpes*, Poland (KF918608) (Wodecka et al. unpublished), and *B. garinii* strain BRZ38 from *I. ricinus* collected in Moravia, Czech Republic (JF828688) (Norek et al. unpublished). In Europe, *B. garinii* follows *B. afzelii* as the most prevalent *B. burgdorferi* s.l. genospecies (Reye et al. 2010). In Poland, the prevalence of *B. garinii* in infected ticks ranged between 10.1 and 21.4 % (Cisak et al. 2006; Kiewra et al. 2014; Stańczak et al. 2000; Strzelczyk et al. 2006).

Rickettsiae were another pathogen identified in *I. ricinus* in this study. Their presence was confirmed by detection of the specific fragments of *gltA* and 16S rRNA gene, but no positive results were obtained when using primers targeting a fragment of the *ompA* gene. This suggested that detected rickettsiae belonged to the species *R. helvetica* as it is one of the few SFG rickettsiae in which *ompA* gene is not amplified (Roux et al. 1996; Parola et al. 1998). Further sequencing of *gltA* fragment enabled definitive identification. The sequence was 100 % comparable with sequences of *R. helvetica* isolated from *I. ricinus* ticks along Europe, from France (KF447530), Germany (JX627379, strain 4TI3; KC007126, isolate 6DI76), Romania (JX040636; strain 99Bc/Romania) to Russia (AM418450, isolate 1–97). Moreover, the consensus sequence of the 16S rRNA gene was identical to *R. helvetica* clone CsFC (GQ413963) isolated from human cerebrospinal fluid (Påhlson, unpublished), *Rickettsia* sp. IP1 isolated from ticks in Japan (AF 394904, AF394905) (Fournier et al. 2002), and *R. helvetica* strain C9P9 from France (L36212) (Roux and Raoult 1995). *R. helvetica* is widely distributed in the tick population in Poland with the mean prevalence up to 10.6 % (Chmielewski et al. 2009; Stańczak et al. 2008) and was also noted in ticks feeding on deer hosts (10.8–19.0 %) (Stańczak et al. 2009).

It is significant, however, this is the first time *R. helvetica* and *B. garinii* have been noted in *I. ricinus* collected from bats. Furthermore, this is the first PCR positivity to the one of genospecies of *B. burgdorferi* s.l. and SFG *Rickettsia* spp. not only in the *I. ricinus* attached to bats but also in the all hard tick species collected from bats. Thus, *I. ricinus* might be the reservoir for these disease agents not only among terrestrial vertebrates, birds, and humans but also bats. However, since this species rarely parasitizes bats (8 specimens per ca. 4000 ticks picked from bats—0.2 %), it does not play a significant role in the pathogen transmission among bats compared to other vertebrates.

These results have expanded our knowledge about tick vectors and provide a platform for further observation and more detailed studies on the possible role of ticks and other ectoparasites as the vectors of TBD in bats.
